# A comparison of two biological markers of recent hepatitis C virus (HCV) infection: implications for the monitoring of interventions and strategies to reduce HCV transmission among people who inject drugs

**DOI:** 10.2807/1560-7917.ES.2018.23.47.1700635

**Published:** 2018-11-22

**Authors:** Vivian D Hope, Ross J Harris, Peter Vickerman, Lucy Platt, Justin Shute, Katelyn J Cullen, Samreen Ijaz, Sema Mandal, Fortune Ncube, Monica Desai, John V Parry

**Affiliations:** 1 Public Health Institute, Liverpool John Moores University, Liverpool, United Kingdom; 2 National Infection Service, Public Health England, London, United Kingdom; 3 University of Bristol, Bristol, United Kingdom; 4 Faculty of Public Health and Policy, London School of Hygiene and Tropical Medicine, London, United Kingdom; 5 The National Institute for Health Research (NIHR) Health Protection Research Unit (HPRU) in Blood Borne and Sexually Transmitted Infections at University College London, London, United Kingdom

**Keywords:** hepatitis C, incidence, monitoring, people who inject drugs, intervention impact

## Abstract

**Background:**

Monitoring hepatitis C virus (HCV) incidence is important for assessing intervention impact. Longitudinal studies of people who inject drugs (PWID), using repeated biological tests, are costly; alternatively, incidence can be estimated using biological markers of recent infection in cross-sectional studies.

**Aim:**

We aimed to compare incidence estimates obtained from two different biological markers of recent infection in a cross-sectional study to inform monitoring approaches for HCV elimination strategies.

**Method:**

Samples from an unlinked anonymous bio-behavioural survey of PWID were tested for two recent infection markers: HCV RNA with anti-HCV negative (‘RNA’) and low-avidity anti-HCV with HCV RNA present (‘avidity’). These two markers were used separately and in combination to estimate HCV incidence.

**Results:**

Between 2011 and 2013, 2,816 anti-HIV-negative PWID (25% female) who had injected during the preceding year were either HCV-negative or had one of the two markers of recent infection: 57 (2.0%) had the RNA marker and 90 (3.2%) the avidity marker. The two markers had similar distributions of risk and demographic factors. Pooled estimated incidence was 12.3 per 100 person-years (pyrs) (95% credible interval: 8.8–17.0) and not significantly different to avidity-only (p = 0.865) and RNA-only (p = 0.691) estimates. However, the RNA marker is limited by its short duration before anti-HCV seroconversion and the avidity marker by uncertainty around its duration.

**Conclusion:**

Both markers have utility in monitoring HCV incidence among PWID. When HCV transmission is high, one marker may provide an accurate estimate of incidence; when it is low or decreasing, a combination may be required.

## Introduction

In high-income countries, hepatitis C virus (HCV) transmission is focused among people who inject drugs (PWID), with infection often acquired soon after initiation to injecting [1,2]. In the United Kingdom (UK), it is estimated that around half of PWID have been infected with HCV [3], although there is considerable geographical variation in prevalence [4]. Estimates of HCV incidence among PWID are high, typically between 10 and 20 infections per 100 person-years (pyrs) of exposure [5].

Measuring incidence, and how this changes over time, is important for assessing the impact of interventions to prevent and control HCV, such as needle and syringe programmes (NSPs), opioid substitution therapy (OST) and HCV treatment as prevention (HCV TasP), as well as monitoring progress towards the global goal of eliminating HCV as a major public health threat [6]. Incidence is traditionally estimated through longitudinal follow-up studies; however, such studies are expensive and difficult among PWID because follow-up is hindered by the illicit nature of drug use and the marginalisation of PWID [7]. An alternative approach is to use biological markers of recent infection in cross-sectional studies. Two approaches have been advocated.

The first approach involves the detection of HCV RNA among individuals found to be HCV antibody (anti-HCV)-negative, indicating an acute and therefore incident infection [8]. Although evidence indicates the possibility of some misclassification [9,10], the length of time that someone remains in this state after infection (the ‘window period’) is short (< 2 months [9,11,12]) and therefore large sample sizes are needed unless incidence is very high.

The second approach is antibody avidity [13,14], an approach that has been used with some success for HIV and acute hepatitis B infections [15,16]. Samples with low avidity anti-HCV are typically indicative of a recent primary infection. The distribution of the window period has been assessed in panels of HCV-infected individuals with a known seroconversion date. Unfortunately, there is uncertainty surrounding the duration of the low-avidity period, with available evidence suggesting it is between 2 and 6 months [17-19]. In addition, some individuals may continue to have weak avidity even when the infection is fully established, as demonstrated for HIV [20], or if antibody titre wanes following spontaneous clearance. Consequently, without appropriate safeguards to exclude cleared infections, the avidity marker may suffer from lower specificity because of false positives.

Both these cross-sectional approaches, as well as cohort-based studies, have been used to measure HCV incidence among PWID in the UK. Two cohort studies suggested considerable variation in incidence from nine per 100 pyrs in South Wales (2004–06) [21] to 42 per 100 pyrs among young PWID in London (2001–02) [22]. Studies examining HCV RNA positivity among anti-HCV-negative PWID produced a similar range of estimates (three to 40 per 100 pyrs) in different cities [7], while a further study using anti-HCV avidity testing indicated that incidence was between four and 12 infections per 100 pyrs across the UK (excluding Scotland) [13]. The marked difference in these UK estimates of HCV incidence among PWID may reflect the fact that studies were undertaken at different times, at a range of locations and/or in populations engaging in different risks, as well as the different incidence measures used.

In order to better understand the determinants underlying the variation in HCV incidence among PWID, we undertook an analysis to compare the estimated incidence, and associated risk factors, for the two different approaches of estimating incidence using biological markers (HCV RNA in antibody-negative individuals and antibody avidity in antibody-positive individuals with HCV RNA) using pooled data from a large national bio-behavioural survey. We also estimate overall incidence based on the two approaches accounting for the uncertainty in their window periods. The findings are important for informing the choice of optimal method for monitoring HCV incidence among PWID in the UK and elsewhere.

## Methods

### Survey

PWID across England, Wales and Northern Ireland are recruited into an annual cross-sectional, unlinked anonymous bio-behavioural survey (the UAM Survey); methodological details have been previously reported [23,24]. In brief, people who have ever injected drugs are recruited through specialist services for PWID providing advice, NSPs, OST or addiction treatment. Service selection reflects the range of services provided for PWID and what is known about geographic variations in drug use. Those agreeing to participate self-complete a short questionnaire and provide a dried-blood spot (DBS) sample at the collaborating service. DBS collection involves obtaining a few drops of blood, through a lancet prick to the finger, onto absorbent filter paper (PerkinElmer 226). The survey has multi-site ethical approval.

In addition to core demographics (age and gender), the questionnaire collects self-reported behavioural data, including prior imprisonment and homelessness, types of psychoactive drug used, injecting risks, uptake of health services (e.g. OST), and sexual behaviours (e.g. condom use). In this study, we included only individuals recruited between 2011 and 2013 inclusive who had injected during the year preceding survey participation.

### Testing for recent infection markers

The DBS samples were tested for antibodies to HIV (anti-HIV), hepatitis C (anti-HCV) and hepatitis B core antibody (anti-HBc). All laboratory testing was carried out at the Virus Reference Department at Public Health England, Colindale, using previously reported methods [8,13]. Two methods were applied to identify recent infections: anti-HCV avidity testing algorithm (upon receipt at laboratory) and RNA testing of the anti-HCV negative samples (on stored samples, with testing undertaken during 2014–15). A 6 mm spot was used for serological and molecular testing.

#### Anti-HCV avidity testing

The method was undertaken as previously described [13,18]. Briefly, each sample is tested in duplicate with one well incubated with urea (avidity well) and the second with wash buffer (control well). In the presence of urea, low-avidity (weakly bound) antibodies will dissociate from HCV antigen bound to the solid phase. An avidity index (AI) is determined for each specimen ((optical density (OD) urea-treated/OD untreated) × 100), with an AI ≤ 40% considered to be low. As low avidity antibody can also be found in individuals who have cleared HCV RNA, specimens with low AI were subsequently tested for HCV RNA using PCR [13]. Those individuals with DBS containing both low-avidity anti-HCV and HCV RNA were considered to have recently acquired their HCV infection (i.e. to have markers compatible with recent primary infection).

#### HCV RNA testing of those anti-HCV-negative

To identify those participants whose samples were anti-HCV-negative and HCV RNA-positive, stored residual DBS samples from those found to be anti-HCV-negative when tested on receipt underwent retrospective HCV RNA testing.

RNA testing involved elution from the DBS by incubating the 6 mm spot for 2 hours at 56 °C with 20 µL of proteinase K and 300 µL of ATL lysis buffer (Qiagen products: 19133 and 19076). The entire eluate was extracted on the Qiagen Biorobot MDX platform using the QIAamp One-For-All Nucleic Acid Kit (Qiagen product: 965672) and One For All MDx cV70a protocol. Brome mosaic virus (BMV) was added as the internal control. Amplification and detection of HCV RNA and BMV was undertaken as previously described [25], employing a PCR targeting the non-coding region of the HCV genome. 

**Figure 1:**
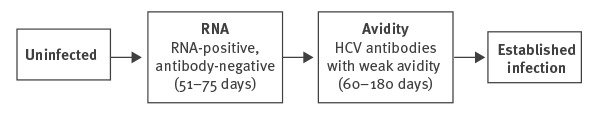
Classification of hepatitis C virus infection status according to RNA and antibody positivity and avidity index of antibody test

These two approaches identified those recently infected at two different stages, which are mutually exclusive, in the sequence from being uninfected to having established infection (Figure 1).

HCV: hepatitis C virus.

Average durations for remaining in recent infection state (the ‘window periods’) are shown in parentheses.

Samples that were anti-HIV positive (n = 25) were excluded, as the effects of HIV on the immune system is likely to affect anti-HCV avidity [20]. Recent HCV infection status was based on the two markers, (i) RNA-positive antibody-negative (henceforth ‘RNA’), and (ii) HCV antibodies with weak avidity in those with HCV RNA (henceforth ‘avidity’). The analytical dataset consisted of individuals who had one of these two markers of recent infection or who were susceptible to HCV infection (uninfected, defined as anti-HCV- and RNA-negative); i.e. those with established infection were excluded. Analysing such data via logistic regression-type models will therefore provide odds ratios (OR) for recent infection according to the two markers.

### Statistical analysis

Patterns of recent infection were examined according to a number of demographic, geographic and risk factor covariates. The demographic variables were year of test (2012 and 2013 vs 2011), age (<30 and ≥40 vs 30–39) and gender (female vs male). The geographic variable was regional prevalence groups that were based on overall HCV antibody prevalence from 2011 to 2013, with low defined as <40% (South West, North East, Wales and Northern Ireland); medium as 40–55% (East of England, West Midlands, East Midlands) and high as >55% (London, North West, South East, Yorkshire and the Humber). The risk factor variables were years since first injection (<1, 1–5, 11–14, 15–19 and ≥20 vs 6–10), homelessness (‘yes, not in past year’ and ‘yes, in past year’ vs never), imprisonment (1–4 times and 5 or more times vs never), injecting crack in the past month (yes vs no/did not inject in past month/unknown), injecting speed in the past month (yes vs no/did not inject in past month/unknown) and injecting and equipment sharing behaviour in the past month (derived from two sequential questions). The latter group was defined as *did not inject in past month*, *injected in past month but did not share equipment*, and *injected and shared equipment in past month,* and therefore incorporates the difference in risk between those that injected in the past month and those that did not.

A combined outcome for recent infection according to either the RNA or avidity measure was examined in relation to the factors above via logistic regression to estimate ORs for any marker of recent infection vs susceptible. In addition, the RNA and avidity measures of recent infection were each analysed as distinct outcomes (vs those susceptible) in a multinomial logistic model; this allowed the effect of the covariate for the separate outcomes to be modelled in terms of relative risk ratios (RRR). The multinomial model allowed for testing the equivalence of parameters in the model for the two recent infection markers, i.e. whether the two markers provided consistent estimates of the risk factors that are predictive of recent infection. In this context, RRRs and ORs are comparable; in particular, if RRRs for the two markers are identical they will be exactly equal to the OR for the combined outcome.

Univariable analyses were conducted and a multivariable model constructed on the basis of backwards stepwise variable selection with a p value of 0.2 for removal. Variables were selected for the logistic and multinomial logistic models and any variables retained in either model were included in the final set of variables. Analyses were conducted using Stata version 13.1 (StataCorp, College Station, Texas, United States).

We estimated incidence based on markers of recent infection using the formula: *I* = *r*/*wn,*

where *I* is the incidence rate, *w* the length of the window period, *r* the number of individuals with the marker of recent infection and *n* the number not infected [26]. The uncertainty arising from both sampling variability of the binomial data (*r, n*) and the length of the window period were accounted for using a fully Bayesian approach. We specified uniform priors for the window period, ranging from 51 to 75 days for RNA and from 60 to 180 days for avidity. For the latter, we also examined a semi-informative beta(2,2) distribution across the range of the 60–180 day window period, such that the interquartile range of the prior distribution was 99–141 days compared with 90–150 under a uniform prior. Medians of the posterior distributions were taken as point estimates and the 2.5th and 97.5th percentiles as 95% credible intervals (CrI). The model was implemented in WinBUGS version 1.4.3 (Medical Research Council, UK).

## Results

Between 2011 and 2013, there were 2,816 anti-HIV negative participants who had injected during the preceding year whose samples were either anti-HCV-negative or had one of the two markers of a probable recent HCV infection. Of these, 57 (2.0%) were HCV RNA-positive and anti-HCV-negative (‘RNA’) and a further 90 (3.2%) had weak anti-HCV avidity in the presence of HCV RNA (‘avidity’). Overall, the mean age of the participants was 34 years (median: 34; interquartile range: 28–39) and 717 (25%) were female. Table 1 shows numbers of individuals with each marker of infection according to survey year, demographics and risk factor variables.

**Table 1:** Markers of recent hepatitis C infection^a^ according to survey year, demographics and risk factor variables, among people who inject drugs, England, Wales and Northern Ireland, 2011–13 (n = 2,816)

Variable	Category	N	Combined markers of recent infection		RNA marker of recent infection		Avidity marker of recent infection	
			Positive	%	Positive	%	Positive	%
Year	2011	791	33	4.2	15	1.9	18	2.3
	2012	969	58	6.0	18	1.9	40	4.1
	2013	1,056	56	5.3	24	2.3	32	3.0
Age	<30	957	56	5.9	29	3.0	27	2.8
	30–39	1,277	62	4.9	19	1.5	43	3.4
	≥40	582	29	5.0	9	1.5	20	3.4
Gender	Male	2,099	101	4.8	38	1.8	63	3.0
	Female	717	46	6.4	19	2.6	27	3.8
Regional prevalence	Low (<40%)	1,114	50	4.5	26	2.3	24	2.2
	Medium (40–55%)	768	35	4.6	11	1.4	24	3.1
	High (>55%)	934	62	6.6	20	2.1	42	4.5
Injecting duration	<1 year	157	9	5.7	3	1.9	6	3.8
	1–5 years	783	39	5.0	24	3.1	15	1.9
	6–10 years	591	36	6.1	12	2.0	24	4.1
	11–14 years	499	22	4.4	7	1.4	15	3.0
	15–19 years	457	20	4.4	7	1.5	13	2.8
	≥20 years	329	21	6.4	4	1.2	17	5.2
Homelessness	Never	696	23	3.3	11	1.6	12	1.7
	Yes, not past year	1,063	58	5.4	24	2.3	34	3.2
	Yes, past year	1,007	65	6.5	22	2.2	43	4.3
Imprisonment	Never	1,129	54	4.8	22	1.9	32	2.8
	1–4 times	886	33	3.7	14	1.6	19	2.1
	≥5 times	801	60	7.5	21	2.6	39	4.9
Crack injecting	No	2,268	87	3.8	37	1.6	50	2.2
	Yes	548	60	10.9	20	3.6	40	7.3
Speed (amphetamine) injecting	No	2,335	127	5.4	49	2.1	78	3.3
	Yes	481	20	4.2	8	1.7	12	2.5
NSP use	Never/not in past year	499	25	5.0	9	1.8	16	3.2
	In past year	2,300	121	5.3	48	2.1	73	3.2
Injecting and sharing past month	Did not inject past month	674	18	2.7	10	1.5	8	1.2
	Injected and did not share	1,418	59	4.2	21	1.5	38	2.7
	Injected and shared	724	70	9.7	26	3.6	44	6.1

NSP: needle and syringe programmes.

^a^ Antibody-negative with RNA and weak avidity of antibody.

### Associations between risk factors and markers of recent infection

**Figure 2:**
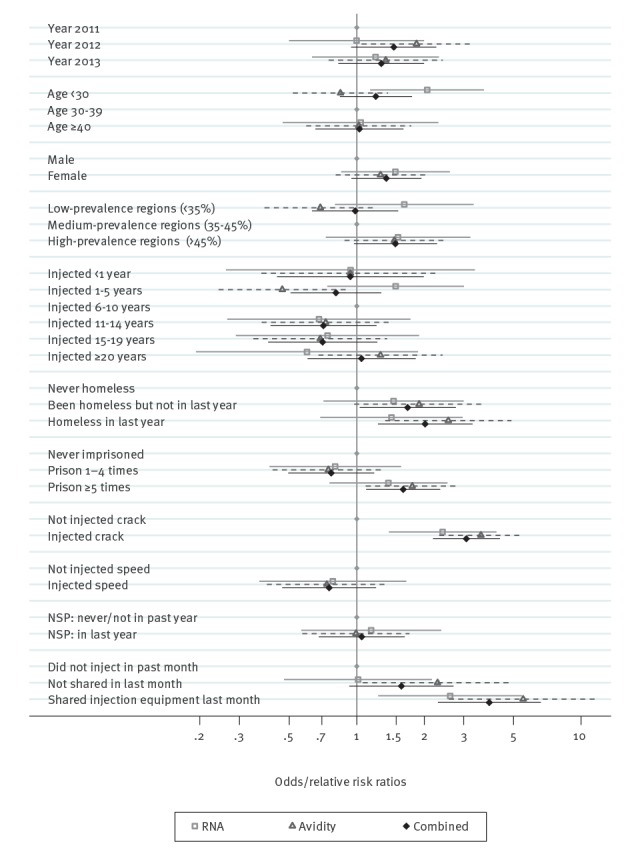
Univariable model results from multinomial logistic models of two markers of recent hepatitis C infection^a^ and logistic regression model for them combined, among people who inject drugs, England, Wales and Northern Ireland, 2011–13 (n = 2,816)

The univariable results from the model (Table 2) are summarised in Figure 2. Year of test and gender were not associated with either measure of incidence. There was evidence of an association (p = 0.03) with age for the RNA marker, with risk highest in those younger than 30 years, but there was no association for the avidity marker (p = 0.042 for inconsistency). There was some evidence of increased risk of recent infection for both markers in high-prevalence areas (OR = 1.49; 95% confidence interval (CI): 0.97–2.28; two markers combined).

**Table 2:** Univariable model results from multinomial logistic model of markers of recent infection^a^ and logistic regression model of combined outcome, among people who inject drugs, England, Wales and Northern Ireland, 2011–13 (n = 2,816)

Variable	Category	Logistic model		Multinomial logistic model				
		Combined OR (95% CI)	p value	RNA RRR (95% CI)	p value	Avidity RRR (95% CI)	p value	p value diff^b^
Year	2011	1 (ref)	0.235	1 (ref)	0.775	1 (ref)	0.088	0.293
	2012	1.46 (0.94–2.27)		1.00 (0.50–1.99)		1.85 (1.05–3.25)		
	2013	1.29 (0.83–­2.00)		1.21 (0.63–2.33)		1.35 (0.75–2.42)		
Age	<30	1.22 (0.84­–1.77)	0.555	2.06 (1.15–3.69)	0.030	0.85 (0.52–1.38)	0.757	0.042
	30–39	1 (ref)		1 (ref)		1 (ref)		
	≥40	1.03 (0.65–1.62)		1.04 (0.47–2.31)		1.02 (0.60–1.75)		
Gender	Male	1 (ref)	0.097	1 (ref)	0.162	1 (ref)	0.298	0.671
	Female	1.36 (0.95–1.94)		1.49 (0.85–2.60)		1.28 (0.81–2.02)		
Regional prevalence	Low (<40%)	0.98 (0.63–1.53)	0.060	1.63 (0.80–3.32)	0.388	0.69 (0.39–1.22)	0.013	0.064
	Medium (40–55%)	1 (ref)		1 (ref)		1 (ref)		
	High (>55%)	1.49 (0.97–2.28)		1.53 (0.73–3.21)		1.47 (0.88–2.45)		
Injecting duration	<1 year	0.94 (0.44–1.99)	0.657	0.94 (0.26–3.37)	0.277	0.94 (0.38–2.34)	0.092	0.032
	1–5 years	0.81 (0.51–1.29)		1.49 (0.74–3.01)		0.47 (0.24–0.90)		
	6–10 years	1 (ref)		1 (ref)		1 (ref)		
	11–14 years	0.71 (0.41–1.23)		0.68 (0.27–1.74)		0.73 (0.38–1.40)		
	15–19 years	0.71 (0.40–1.24)		0.74 (0.29–1.90)		0.69 (0.35–1.37)		
	≥20 years	1.05 (0.60–1.83)		0.60 (0.19–1.88)		1.28 (0.68–2.41)		
Homelessness	Never	1 (ref)	0.018	1 (ref)	0.552	1 (ref)	0.016	0.448
	Yes, not past year	1.69 (1.03–2.76)		1.46 (0.71–3.00)		1.90 (0.98–3.69)		
	Yes, past year	2.02 (1.24–3.28)		1.43 (0.69–2.97)		2.56 (1.34–4.89)		
Imprisonment	Never	1 (ref)	0.002	1 (ref)	0.272	1 (ref)	0.005	0.729
	1–4 times	0.77 (0.49–1.20)		0.80 (0.41–1.58)		0.75 (0.42–1.33)		
	≥5 times	1.61 (1.10–2.36)		1.38 (0.76–2.54)		1.77 (1.1– 2.85)		
Crack injecting	No	1 (ref)	< 0.001	1 (ref)	0.020	1 (ref)	0.001	0.727
	Yes	2.44 (1.58–3.78)		2.23 (1.13–4.37)		2.60 (1.48–4.56)		
Speed (amphetamine) injecting	No	1 (ref)	0.279	1 (ref)	0.460	1 (ref)	0.417	0.962
	Yes	0.76 (0.47–1.24)		0.75 (0.36–1.60)		0.77 (0.41–1.44)		
NSP use	Never/not in past year	1 (ref)	0.819	1 (ref)	0.685	1 (ref)	0.979	0.732
	In past year	1.05 (0.68–1.64)		1.16 (0.57–2.38)		0.99 (0.57–1.72)		
Injecting and sharing past month	Did not inject past month	1 (ref)	< 0.001	1 (ref)	0.002	1 (ref)	< 0.001	0.304
	Injected and did not share	1.58 (0.93–2.70)		1.01 (0.47–2.16)		2.29 (1.06–4.94)		
	Injected and shared	3.90 (2.30–6.62)		2.61 (1.25–5.45)		5.52 (2.58–11.81		

CI: confidence interval; NSP: needle and syringe programmes; OR: odds ratio; ref: reference value; RRR: relative risk ratio.

^a^ RNA-positive antibody-negative (RNA marker) and antibody-positive with weak avidity (avidity marker).

^b^ p value diff is the p value for the effect estimates for RNA and avidity being the same.

NSP: needle and syringe programmes.

^a^ Antibody-negative with RNA (RNA marker) and weak-avidity antibody (avidity marker).

95% confidence intervals are indicated by solid and dashed lines.

Patterns of recent infection were inconsistent according to the two markers for injecting duration (p = 0.032 for inconsistency). For the RNA marker, there was a slightly higher rate in those injecting for 1 to 5 years vs 6 to 10 years (RRR = 1.49; 95% CI: 0.74–3.01), longer durations had non-significant lower rates. For the avidity marker, there were significantly lower rates for 1 to 5 years injecting vs 6 to 10 years (RRR = 0.47; 95% CI: 0.24–0.90) and a non-significant small increase for injecting for more than 20 years. For both markers, there was no evidence of a difference in risk for those injecting for less than 1 year.

Homelessness, being imprisoned five or more times and injecting crack all showed a higher risk of recent HCV infection, with consistent estimates for the two markers. Injecting in the past month showed a modest increase in risk, with a significant association for the avidity marker but not the RNA marker, although overall differences for the two markers again had non-significant p values for inconsistency. Sharing injecting equipment in the past month showed a large increase in risk for both markers of recent HCV infection.

**Figure 3:**
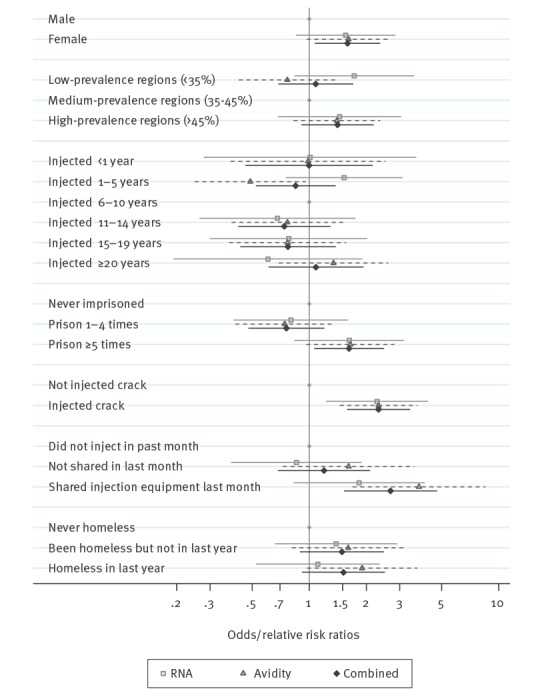
Multivariable model results from multinomial logistic model of markers of recent infection^a^ and logistic regression model of combined outcome, among people who inject drugs, England, Wales and Northern Ireland, 2011–13 (n = 2,816)

^a^RNA positive antibody negative (RNA marker) and antibody positive with weak avidity (avidity marker).

The stepwise selection procedure included gender, region, injecting duration, homelessness, imprisonment, crack injecting and injected/shared injecting equipment in the past month in the final model (Figure 3 and Table 3). The ORs and RRRs were generally similar to the univariable results (Table 2), but risk factors with stronger associations were attenuated somewhat, such as homelessness (adjusted odds ratio (AOR) = 1.52 vs OR = 2.02 for homeless in last year, two markers combined) and sharing injecting equipment in the past month (AOR = 2.69 vs OR = 3.90, two markers combined). Interestingly, gender showed some evidence of an association in the adjusted model (AOR = 1.59; 95% CI: 1.07–2.37, two markers combined) but not in the univariable models.

95% confidence intervals are indicated by solid and dashed lines.

**Table 3:** Multivariable model results from multinomial logistic model of markers of recent infection^a^ and logistic regression model of combined outcome, among people who inject drugs, England, Wales and Northern Ireland, 2011–13 (n = 2,816)

Variable	Category	Logistic model		Multinomial logistic model				
		Combined OR (95% CI)	p value	RNA RRR (95% CI)	p value	Avidity RRR (95% CI)	p value	p value diff^b^
Gender	Male	1 (ref)	0.022	1 (ref)	0.152	1 (ref)	0.072	0.962
	Female	1.59 (1.07–2.37)		1.55 (0.85–2.84)		1.61 (0.97–2.68)		
Regional prevalence	Low (<40%)	1.08 (0.69–1.71)	0.237	1.73 (0.83–3.57)	0.339	0.77 (0.42–1.38)	0.086	0.104
	Medium (40–55%)	1 (ref)		1 (ref)		1 (ref)		
	High (>55%)	1.41 (0.91–2.18)		1.45 (0.68–3.06)		1.40 (0.83–2.37)		
Injecting duration^c^	<1 year	1.00 (0.46–2.16)	0.790	1.01 (0.28–3.68)	0.309	0.99 (0.38–2.53)	0.129	0.032
	1–5 years	0.85 (0.52–1.38)		1.53 (0.75–3.12)		0.49 (0.25–0.96)		
	6–10 years	1 (ref)		1 (ref)		1 (ref)		
	11–14 years	0.74 (0.42–1.29)		0.68 (0.26–1.75)		0.77 (0.39–1.51)		
	15–19 years	0.77 (0.43–1.38)		0.78 (0.30–2.02)		0.77 (0.38–1.57)		
	≥20 years	1.09 (0.61–1.93)		0.61 (0.19–1.92)		1.34 (0.69–2.61)		
Homelessness	Never	1 (ref)	0.237	1 (ref)	0.631	1 (ref)	0.103	0.234
	Yes, not past year	1.49 (0.89–2.48)		1.38 (0.66–2.91)		1.61 (0.81–3.20)		
	Yes, past year	1.52 (0.92–2.51)		1.11 (0.52–2.36)		1.90 (0.97–3.71)		
Imprisonment	Never	1 (ref)	0.003	1 (ref)	0.122	1 (ref)	0.019	0.974
	1–4 times	0.76 (0.48–1.20)		0.80 (0.40–1.61)		0.74 (0.41–1.35)		
	≥5 times	1.62 (1.06–2.48)		1.63 (0.83–3.17)		1.65 (0.96–2.81)		
Crack injecting	No	1 (ref)	< 0.001	1 (ref)	0.009	1 (ref)	< 0.001	0.949
	Yes	2.32 (1.58–3.41)		2.28 (1.22–4.24)		2.32 (1.45–3.72)		
Injecting and sharing past month	Did not inject past month	1 (ref)	< 0.001	1 (ref)	0.036	1 (ref)	< 0.001	0.438
	Injected and did not share	1.20 (0.68–2.10)		0.86 (0.39–1.89)		1.61 (0.73–3.59)		
	Injected and shared	2.69 (1.52–4.73)		1.83 (0.83–4.06)		3.79 (1.69–8.50)		

CI: 95% confidence interval; OR: odds ratio; ref: reference value; RRR: relative risk ratio.

^a^ RNA-positive antibody-negative (RNA marker) and antibody-positive with weak avidity (avidity marker).

^b^ p value diff is the p value for the effect estimates for RNA and avidity being the same.

^c^ Using three duration categories 0–5, 6–14 and ≥15 years, with 6–14 years as baseline, the pattern is similar. For the 0–5 years category for RNA, RRR = 1.64 (95% CI: 0.91–2.98) and for avidity, RRR = 0.80 (95% CI: 0.38–1.70). For the ≥15 years category for the RNA, RRR = 0.62 (95% CI: 0.36–1.07) and for avidity, RRR = 1.07 (95% CI: 0.66–1.73).

### Estimated incidence

The pooled estimate of incidence from the Bayesian model was 12.3 per 100 pyrs (95% CrI: 8.8–17.0) when using both markers of recent infection. As expected, the posterior distribution for the window period of RNA was near identical to the prior, i.e. uniformly distributed between 51 and 75 days. However, the posterior distribution for the weak-avidity window was shifted somewhat from the prior distribution to be consistent with the RNA marker data, with a median duration of 100 days (vs a mid-point of 120 days) and a 95% CrI of 68 to 148 days. Using RNA alone gave an incidence estimate of 12.7 per 100 pyrs (95% CrI: 9.1–17.7), very similar to the pooled estimate (p value for difference = 0.891). Using the avidity marker alone gave an incidence estimate of 11.8 per 100 pyrs (95% CrI: 6.6–21.3); this was imprecise owing to the uncertainty about the duration of the low-avidity period. With a semi-informative beta distribution for the window period, the estimate was 11.1 per 100 pyrs (95% CrI: 7.0–18.9), a modest improvement in precision; this made no difference in the combined model. The avidity-only estimate (with informative window) was not significantly different to the pooled estimate (p = 0.865) or from the RNA-only estimate (p = 0.691).

## Discussion

Our study is unique in comparing two biological markers of recent HCV infection in a large sample of community-recruited PWID. The factors associated with the recent infections identified by combining both markers, HCV RNA in those anti-HCV negative and HCV antibody avidity, were similar to those identified by each measure separately, and were generally expected associations with known HCV risk factors. In our combined model using both markers, the avidity marker contributed little information on absolute incidence rates owing to substantial uncertainty over the window period for this measure, with the combined result driven by the RNA data and its more certain window period. Nevertheless, incidence estimates from the two measures were very close, and the pooled analysis may give a better idea of the true window period for weak avidity, which we estimated at around 100 days on average, although 95% CrI were still wide, ranging from 68 to 148 days.

The factors considered here in relation to recent infection were a mixture of indicators of elevated risk, such as frequency of imprisonment [13], and risk behaviours representing a recent infection risk, such as sharing injecting equipment [21,27,28]. We found consistent patterns for the two markers (and when combined), with factors that have previously been shown to be associated with increased risk of HCV infection: female gender [28-31], imprisonment [13,32], the injection of crack [22,28] and the sharing of injecting equipment [33,34]. Results for injecting duration showed some inconsistency but in general, factors that showed a strong association with recent infection had consistent results for both markers.

Previous studies of risk factors for HCV and modelling work, including force of infection (FOI) estimates, have generally indicated an increased infection risk during the first year of injecting [28,35], in those who started injecting recently [27,28] or who had been injecting for only a few years [36]. We did not find such an association; however, our data are somewhat sparse, with just nine recent infections (i.e. having the RNA or avidity marker) in those injecting for less than one year; nevertheless, there should be sufficient power to detect a threefold or higher increase in risk. It is possible that some respondents imprecisely recalled their age at first injection, which would have led to an incorrect time since first injection and thereby some misclassification of recent infections. Alternatively, high excess risk at initiation may largely be due to the first few injecting events, when the individual may not have learned to inject themselves or started to use services [37], leading to these being under-represented in our service based sample. While our survey approach is established, the illicit and marginalised nature of injecting drug use makes construction of a formal sampling frame impossible.

The UK’s mature epidemics of injecting drug use and HCV have resulted in an ageing population of PWID with a stable HCV prevalence [38]. This could possibly result in a different pattern of HCV incidence with time since first injection compared with that found, for example, in an immature injecting epidemic with many recent initiates or where HCV prevalence among PWID is very high. It is possible that re-initiation to injecting after periods of cessation, for example during or after addiction treatment or imprisonment [39,40], may result in repeated short periods of elevated risk throughout a lifetime of injecting, similar to that which probably occurs at first initiation. Although this needs further investigation, the association with imprisonment, found here and previously noted [13,32], supports this possibility.

A related issue, although not significant, was that for the avidity marker, a higher proportion of the recent infections detected were among those who first injected more than 20 years ago (19% vs 7% for RNA). This might reflect misclassification bias and possibly be due to a small number of people with longer-term infections not developing HCV antibodies with high avidity. Reported false recency rates are less than 1–2% in individuals without HIV [14]. However, there may still be issues with reduced immune response in those with long-term HCV infection, similar to those seen in individuals living with HIV [20], but related to other co-morbidities.

Misclassification or non-capture of recent initiates would be expected to reduce the number of recent infections observed in this group, but as the vast majority of participants had been injecting for longer, this should have a relatively small impact on the overall incidence. Further, this is a minor point compared with the uncertainty of the window periods themselves [8,11,17,18] and does not preclude the use of recent infection markers to monitor trends in incidence. However, further work, for example using seroconversion panels, to improve understanding of the window periods would be helpful and is needed before routine clinical use of avidity testing.

Owing to the uncertainties in the window period for weak avidity, the combined model for incidence relied largely on the RNA data. Nevertheless, both markers have their merits: RNA is a reliable and well-understood marker of recent infection, but infrequently observed because of the short window period. The avidity-based marker may not be ideal for estimating absolute incidence, but has greater power to detect patterns according to risk factors or changes over time. By combining both markers, we could identify risk factors for recent infection for which there was insufficient power – even in our relatively large sample – when using either marker alone. Future work could incorporate markers of recent infection and the FOI approach within a combined model. Although work is required to resolve a number of issues, such as the discrepancy between biological markers and FOI methods for injecting duration-specific risk, such modelling has the potential to simultaneously refine window period estimates and provide estimates of incidence with greater power.

The combined use of both markers of recent infection could reduce the need for very large sample sizes and costs. Power calculations using a simple simulation-based approach [41] indicate that a survey recruiting annually around 1,000 participants without an established infection, and where around 5% of these participants had one of the two markers of recent infection at baseline, there would be over 90% power of detecting a halving of incidence over 4 years, although a reduction by one third may not be detectable (56% power). If the RNA marker is used alone (ca 2% at baseline), only a reduction by two thirds would be reliably detected (power = 86%) and detecting a halving of incidence is not guaranteed (power = 54%). This is a general problem with detecting decreases in incidence as over time, the number of recent infections will become extremely low. Of course, a genuine decrease in incidence will also result in lower prevalence, especially in recent initiates to injecting; therefore a combined approach may be most useful for detecting the impact of TasP, NSP and OST, and in monitoring progress towards the global strategy target to reduce HCV incidence by 90% by 2030 [6]. However, the actual approach taken in a country or area will depend on the baseline hepatitis C incidence and the rate of decline expected. These approaches will usually provide a better insight into the extent of recent infections than data on new HCV diagnoses, as people may have been infected some time before their diagnosis and changes in diagnosis rates may not reflect underlying incidence but rather detection rates and testing practice, e.g. following temporal variations in the offer and uptake of diagnostic testing. However, monitoring incidence using the approaches applied here may not be practical among those populations and groups where the overall HCV incidence and risks are very low; very large sample sizes would be needed, and combination of methods may therefore be required.

## Conclusion

Our findings indicate that the two biological approaches to estimating incidence identified associations with similar injecting risk behaviours and social and demographic profiles. This indicates that both have utility in monitoring incidence, although the short-lived nature of the states assessed by these two markers limits the use of only one marker, particularly when the incidence is not high. Meeting the hepatitis C elimination goals in the Global Health Sector Strategy for Hepatitis [6], will require an assessment of the impact on HCV transmission of interventions such as NSPs, OST and TasP that need to be delivered to PWID to achieve it, in the context of declining incidence. A robust measure of incidence among PWID could be provided by using the two biological markers examined here in combination.
